# Differential Tolerance to Lead and Cadmium of Micropropagated *Gypsophila fastigiata* Ecotype

**DOI:** 10.1007/s11270-018-3702-8

**Published:** 2018-01-25

**Authors:** Ewa Muszyńska, Ewa Hanus-Fajerska, Aleksandra Koźmińska

**Affiliations:** 1 Department of Botany, Warsaw University of Life Sciences (SGGW), Faculty of Agriculture and Biology, Nowoursynowska 159, Building 37, 02-776 Warsaw, Poland; 2 Faculty of Biotechnology and Horticulture, Institute of Plant Biology and Biotechnology, Unit of Botany and Plant Physiology, University of Agriculture, al. 29-Listopada 54, 31-425 Krakow, Poland

**Keywords:** Cadmium, Facultative metallophyte, In vitro selection, Lead, Medium supplementation

## Abstract

In vitro techniques may provide a suitable tool for effective propagation and conservation of plant species representing various ecological niches. The elaboration of such protocols is also prerequisite for selection of heavy-metal-tolerant plant material that could be afterwards used for restoration or remediation of polluted sites. In this study, culture protocol for *Gypsophila fastigiata* propagation was developed. The highest multiplication coefficient, which reached 6.5, and the best growth parameters were obtained on modified MS medium supplemented with 1.0 mg L^−1^ 2iP and 0.2 mg L^−1^ IAA. The obtained cultures were treated with different concentrations of lead nitrate (0.1, 0.5, and 1.0 mM Pb(NO_3_)_2_) or cadmium chloride (0.5, 2.5, and 5.0 μM CdCl_2_). The growth parameters, photosynthetic pigments, and phenolic compound content were examined in order to evaluate whether tested metal salts can have an adverse impact on studied culture. It was ascertained that Pb ions induced growth disturbances and contributed to shoot wither. On the contrary, the proliferative shoot cultures were established on media containing Cd ions and the multiplication coefficients and shoot length increased on all media enriched with CdCl_2_. Chlorophylls and carotenoid contents were negatively affected by application of 5.0 μM of cadmium; nevertheless, in shoots treated with 2.5 μM CdCl_2_, increased accumulation of photosynthetic pigments occurred and their amount was similar to untreated culture. Adaptation to Cd was associated with stimulation of phenolic compound synthesis. Hence, we have reported on unambiguous positive result of in vitro selection procedure to obtain vigorous shoot culture tolerant to cadmium.

**Electronic supplementary material:**

The online version of this article (10.1007/s11270-018-3702-8) contains supplementary material, which is available to authorized users.

## Introduction

For years, metallophytes of both natural and human-influenced metalliferous soils have focused considerable attention due to their unique appearance and ability to colonize extremely harsh habitats. Through evolution, the plants occurring on metalliferous habitats have developed a range of intriguing adaptive traits, demonstrated as unique morphological, behavioral, and physiological alterations that enable them to avoid or tolerate metal toxicity (Gołębiewski et al. 2014; Woch et al. 2016; Wójcik et al. 2017). Thus, metallophytes possess mechanisms responsible for plant cell protection from excess amount of metallic ions which may get into the protoplast as well as for their detoxification inside the cell by chelation, vacuolar sequestration, or exclusion from the symplast. Such mechanisms have resulted in highly specialized plants able to (hyper) accumulate or avoid metals from the shoots. These unique taxa, mainly species or subspecies have the great potential to be applied during rehabilitation schemes and/or phytoremediation of metal-polluted sites. Moreover, metal-rich plant biomass can be used as a “bio-ore” for recovery of precious metallic elements (phytomining, agromining), as an eco-catalysts for various chemical transformations or as a natural source of micronutrients that are essential for human diet (biofortification). Undoubtedly, there is an urgent need of protection of metalliferous sites and conservation of metallophyte biodiversity (Muszyńska et al. 2017; Muszyńska and Hanus-Fajerska 2017; Wójcik et al. 2017). During the last several years, in vitro culture techniques have been extensively developed and applied to support plant conservation (Engelman 2011; Kikowska et al. 2014; Alfonso et al. 2017; Boisson et al. 2017). The elaboration of effective protocols which allow to micropropagate of valuable plant species is also prerequisite for genetic transformation procedures that may improve their potential for environmental remediation (Doran 2009; da Conceição Gomes et al. 2016; Das et al. 2016). A feasible and rather cost-effective alternative to genetic manipulation could be the acquisition of resistant plant material by in vitro selection (Wiszniewska et al. 2015; Kumar et al. 2016). This approach can operate under wholly controlled conditions, with limited space, and can considerably shorten the time needed for searching of desirable traits under given selection pressure with minimal environmental interaction (Ribalta et al. 2014). Thus, it can complement the usual field selection. Plants tolerant to various abiotic and biotic stresses might be obtained by applying the chosen cultivation protocol with appropriate selecting agents such as NaCl (for generating salt tolerance), PEG or mannitol (for drought tolerance), trace metals/metalloids (for heavy metals/metalloids tolerance), pathogen culture filtrate, phytotoxin, or pathogen itself (for disease resistance) (Hanus-Fajerska et al. 2000; Ashrafzzadeh and Leung 2015; Wiszniewska et al. 2015, 2017; El-Minisy et al. 2016; Kumar et al. 2016). Only the explants capable of long run surviving in the presence of proper agent are selected due to the induction of genetic variation among cells, tissues, and/or organs in cultured and regenerated plants (Rai et al. 2001; Sakhanokho and Kelley 2009). In vitro selection might thus efficiently save the time required for developing abiotic stress tolerant or disease resistant line of important plant species but genetic stability of the selected trait should be confirmed, and selected variants should be finally field-tested. Although there are genetic, biochemical, and physiological constraints in obtaining in vitro stress-tolerant plants this technique has been successfully used to produce stress-tolerant plants from several genus (Mohamed et al. 2000; Wiszniewska et al. 2015; Kumar et al. 2016). Therefore, there is not much literature data on tissue culture of metallophytes for this purpose (Zheng et al. 2007; Wiszniewska et al. 2015; Muszyńska et al. 2017).

*Gypsophila fastigiata* (Caryophyllaceae) spontaneously occurs on the Zn-Pb waste heaps located in the south-eastern part of the Śląsko-Krakowska Upland, Poland (Muszyńska et al. 2015; Woch et al. 2016). As local ecotype adapted to unfavorable conditions of toxic heavy metals’ level, nutrient deficiency, high insolation, strong wind, drought, unfavorable pH value, it is a unique plant species. The ex situ conservation might contribute to the maintenance of genetic diversity of the genus. Therefore, in the present experiment, we proposed to elaborate the multiplication protocol of *G. fastigiata* that allows the preservation of these metal-tolerant species. We hypothesized that the calamine ecotype of *G. fastigiata* would require the addition of lead or cadmium ions to the culture medium. Thus, simultaneously, we could undertake the selection of lines tolerant to those heavy metals. Such approach of metallophytes selection by in vitro methods incorporated with molecular and functional genomics can provide a new opportunity to improve stress tolerance in plants relevant to environmental sustainability.

## Materials and Methods

### Source of Plant Material

The donor material to initiate in vitro culture was seed samples taken from *G. fastigiata* L. specimens (Caryophyllaceae Juss.) belonging to the calamine population which spontaneously appears on an old waste heap obtained after Zn-Pb ore mining and processing in the Olkusz Ore-bearing region (south-eastern part of the Śląsko-Krakowska Upland, Poland). The seeds were immersed in 70% (*v*/*v*) ethanol for 1 min and surface decontaminated with 0.05% mercuric chloride for 4 min. After five washes with sterile distilled water, they were put onto MS medium (Murashige and Skoog 1962) without plant growth regulators. Shoot tips of aseptically obtained seedlings were used as primary explants.

### Elaboration of Propagation Protocol

#### Establishment of Proliferating Shoot Culture

Seedling shoots deprived of roots were placed onto MS medium supplemented with 20 g L^−1^ sucrose, 0.65 mg L^−1^ calcium gluconate, 0.5 g L^−1^ polyvinylpyrrolidone (PVP), and 0.5 g L^−1^ 2-N-morpholino-ethanesulfonic acid (MES). The following composition of plant growth regulators added to MS medium were tested:

1. 1.0 mg L^−1^ BAP + 0.2 mg L^−1^ NAA (described further as *D1)*
2. 1.0 mg L^−1^ 2iP + 0.2 mg L^−1^ NAA (described further as *D2)*
3. 1.0 mg L^−1^ BAP + 0.2 mg L^−1^ IAA (described further as *D3)*
4. 1.0 mg L^−1^ 2iP + 0.2 mg L^−1^ IAA (described further as *D4)*

The media were solidified with 0.8% Difco Bacto agar, and their pH was adjusted to 5.8 before autoclaving. Five explants per 100-mL Erlenmeyer flask were explanted on the respective media. Six flasks per each treatment were used, which correspond to 30 explants per 1 replication. Subcultures were done with 8-week intervals. After 16 weeks, the shoots were counted and micropropagation coefficient (MC) was calculated using the following formula, which was previously successfully used by Muszyńska and Hanus-Fajerska (2017) for another taxonomically related species belonging to Caryophyllaceae:

MC = number of induced adventitious shoots/total number of explants

Shoots (as well as roots if developed) were measured and weighted. For dry matter determination, the plant material was oven-dried in 105 °C for 24 h and weighted afterward.

#### The Rooting Stage

In vitro raised shoots cut under laminar flow chamber were used to investigate the rooting efficiency. The effect of modified MS medium differing in the content of macro- and microelements (by supplementation with 20 g L^−1^ sucrose, 0.65 g L^−1^ calcium gluconate, 0.5 g L^−1^ PVP, 0.5 g L^−1^ MES), and with addition of 1 mg L^−1^ IAA, on the rooting stage was verified (described further as *MSR*). The variant of the rooting medium contained all the same ingredients but the macro- and micronutrients were reduced by half (described further as *1/2 MSR*). The media were solidified with 0.8% Difco Bacto agar, and pH was adjusted to 5.8 before autoclaving. Five shoots, about 15–20 mm long, per 100-mL Erlenmeyer flask were explanted on the respective rooting media. The assessment of rooting dynamics was conducted during 8 weeks. Counting of adventitious roots was carried out every 4 days during this period.

#### Acclimatization of Microcuttings

Thirty-five microcuttings rooted on *1/2 MSR* medium were transferred to ceramic pots in diameter of 90 mm filled with sterile mixture of perlite and horticultural soil in 1:1 ratio. During the first 2 weeks, plantlets were protected with transparent containers in order to provide optimum humidity. Afterwards, they were transferred to the greenhouse with a temperature of 18–20 °C. The percentage of survived specimens was calculated after 8 weeks of ex vitro planting, and at that time, they were transplanted to bigger pots (100 mm of diameter) containing a mixture of perlite, horticultural soil, and calamine substratum on which population of examined plant species was grown in natural conditions (1:1:3 *v*/*v*). The chemical properties of calamine substratum were previously characterized in details and described by Muszyńska et al. (2017).

#### In Vitro Selection and Evaluation of Plant Growth Parameters

The selection was conducted using the medium supplemented with different combination of lead nitrate or cadmium chloride. The following treatments were evaluated: 0.1, 0.5, and 1.0 mM Pb(NO_3_)_2_ as well as 0.5, 2.5, and 5.0 μM CdCl_2_. Five explants (microcuttings) per 100-mL Erlenmeyer flask were explanted on the respective media. Six flasks per each treatment were used, which correspond to 30 explants per 1 replication. Subcultures were done with 8-week intervals. After 24 weeks of heavy metals treatment, the obtained cultures were measured and weighted similarly to the previous step related to the optimization of proliferating shoot protocol. Into this step of experimental scheme *D4* medium was applied. The main measure of medium effectiveness was the efficiency of multiplication and fitness of prolonged shoot culture.

#### Biochemical Analysis

At the end of heavy metal treatment, the physiological condition was determined using UV/VIS spectrophotometry (Hitachi U-2900 spectrophotometer, Japan). For all biochemical analyses, three randomly chosen shoot samples (each of 0.1 g) per treatment were used. The content of photosynthetic pigments in obtained plant material was determined according to Wellburn (1994). The samples were ground with 80% acetone with the addition of CaCO_3_. The obtained extract was centrifuged for 15 min at 4800 rpm, and supernatant was filled up to a volume of 10 mL with 80% acetone. The chlorophyll *a*, chlorophyll *b*, and carotenoid contents were estimated by measurement of the absorbance at 470, 646, and 663 nm, respectively. The concentration of phenolic compounds was determined according to Fukumoto and Mazza (2000). The samples were homogenized with 10 mL of 80% methanol and centrifuged for 15 min at 4800 rpm. The supernatant was mixed with 0.1%HCl (in 96% ethanol) and 2 %HCl (in water), and after 15 min, the absorbance at 280, 320, 360, and 520 nm was read for total phenols, phenolic acids, flavonols, and anthocyanins, respectively. Chlorogenic acid (sum of phenols) caffeic acid (phenylpropanoids), quercetin (flavonoids), and cyanidin (anthocyanins) were used to determine the particular group of phenols.

### Experimental Design and Statistical Analysis

In total, the experiment lasted 48 weeks with an 8-week subcultures. Therein, the optimization of multiplication protocol was proceeded during 16 weeks and the rooting stage lasted 8 weeks, while the heavy metal treatment was performed during the subsequent 24 weeks. The experiment was repeated independently three times (three replications). Microcuttings were randomly assigned to treatments. All media were prepared directly before the culture establishment and autoclaved at 121 °C, 0.1 MPa for 20 min. The cultures were maintained in a growth chamber at 24 °C day/20 °C night, under 16-h photoperiod (photon flux density 80 μmol m^2^ s^−1^, Philips TL 33).

The data were subjected to ANOVA analysis (STATISTICA 12.5, StatSoft, Tulsa, OK, USA), and a post hoc Fisher’s test was performed to determine differences between treatments at *P* < 0.05. Some biometrical parameters were evaluated with Pearson’s linear correlation, and correlation coefficients were estimated.

## Results

### Elaboration of Propagation Protocol

**Fig. 1:**
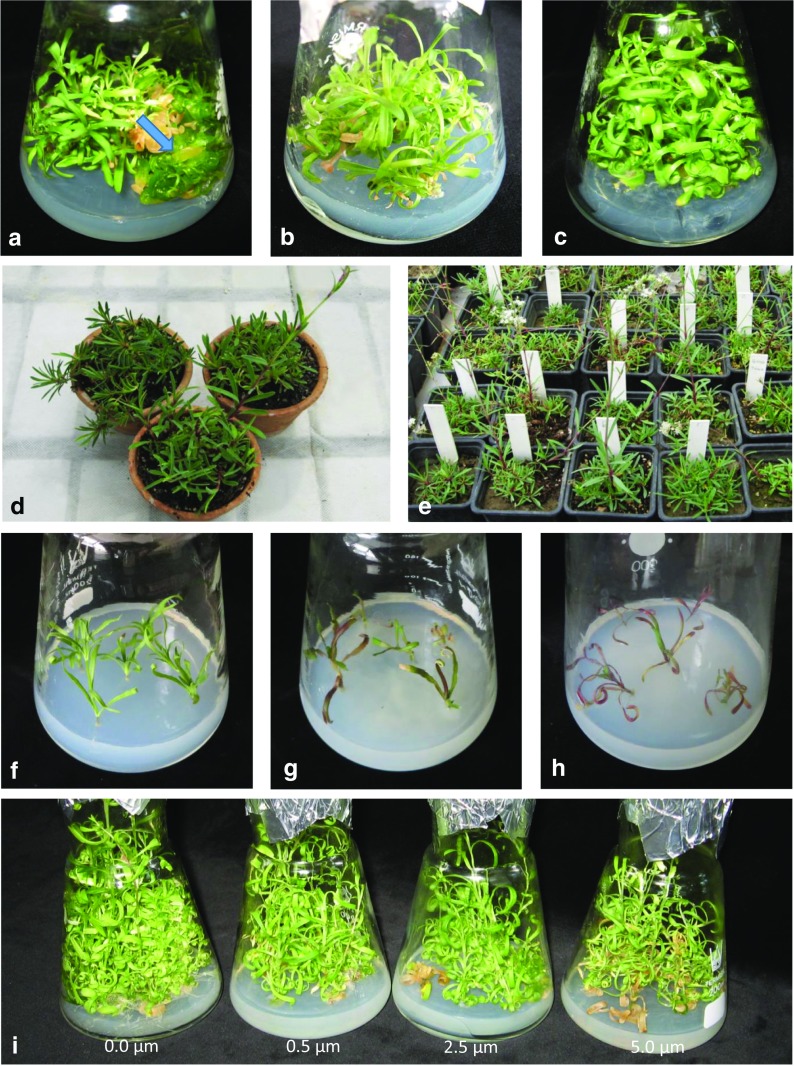
The growth of *G. fastigiata* cultures on various experimental stages. **a**–**c** Proliferative shoot cultures on medium D2 (**a**), D3 (**b**), and D4 (**c**) during optimization of clonal propagation. **d**, **e** Acclimatized microcuttings after 8 weeks of ex vitro growth (**d**) and during their cultivation on calamine substratum (**e**). **f**–**h** Leaves necrosis and shoots dying on medium enriched with 0.1 (**f**), 0.5 (**g**), and 1.0 mM (**h**) Pb(NO_3_)_2_. **i** Culture reaction on increasing concentration of CdCl_2_ in the growth medium

Proliferative shoot cultures were obtained regardless of tested plant growth regulators, and micropropagation coefficient of *G. fastigiata* ranged between 3.5 and 6.5 (Table 1; Fig. 1a–c). The greatest efficiency of shoot multiplication was observed on *D4* medium supplemented with 1.0 mg L^−1^ 2iP and 0.2 mg L^−1^ IAA (MC = 6.5). In comparison with this treatment, statistically significant reduction in the number of regenerated shoots from a single explant was noticed on cultures treated with 1.0 mg L^−1^ BAP and various auxins, i.e., 0.2 mg L^−1^ NAA (*D1* medium) or 0.2 mg L^−1^ IAA (*D3* medium), and amounted MC = 3.8 and MC = 3.5, respectively. Moreover, the shoots regenerated on *D4* medium was almost three times longer than the shortest ones from *D1* medium, and their length reached the highest value, i.e. 27 mm. The differences between treatments in the shoot number and their length resulted in significant variation in shoots fresh matter content. The lowest value, which was about 74% of the highest one obtained on *D4* medium (approx. 1 g), was noted for shoots proliferated on *D1* and *D3* medium (Table 1). Although on *D2* medium the values of all examined biometric parameters were found to be intermediate, the regenerated shoots were thick, sometimes vitreous and curled (Fig. 1a).

**Table 1:** The influence of different media composition on *G. fastigiata* growth parameters after 16 weeks of in vitro cultivation

Culture medium	Multiplication coefficient	Shoot length (mm)	Shoot fresh weight (g)	Shoot dry weight (% f.w.)	No. of roots/explant	Root length (mm)	Root fresh weight (g)	Root dry weight (% f.w.)
*D1*	3.83 bc^a^	9.85 c	0.745 c^a^	8.57 b	0.00	0.00	0.000	0.00
*D2*	5.29 ab	14.35 b	0.838 b	7.06 c	0.00	0.00	0.000	0.00
*D3*	3.50 bc	12.53 bc	0.743 c	10.13 a	0.00	0.00	0.000	0.00
*D4*	6.50 a	27.10 a	1.045 a	7.11 c	2.71	48.96	0.102	8.16

Means indicated by the same letter within the columns do not significantly differ at *P* < 0.05 according to Fisher’s test

^a^Values are means of three replicates

**Fig. 2:**
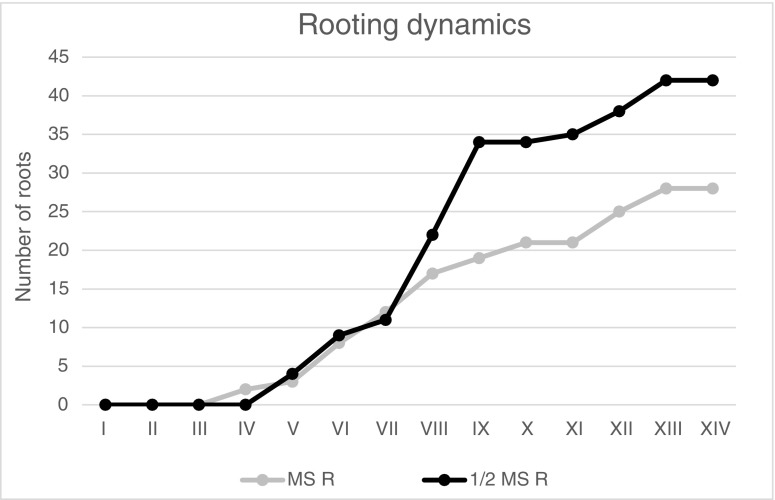
Root growth dynamics of *G. fastigiata* on media differing in the content of macro- and microelements. The following observations (I–XIV) were performed every 4 days for 8 weeks

The applied combination of plant growth regulators did not stimulate the spontaneous regeneration of adventitious roots (Table 1). The exception was *D4* medium enriched with 1.0 mg L^−1^ 2iP and 0.2 mg L^−1^ IAA on which rhizogenesis was noticed. Nevertheless, the values of examined rooting characteristic, such as roots number/explant, their length, as well as fresh and dry matter were not very satisfactory. For this reason, in the elaborated clonal propagation separate rooting stage was indeed necessary. The rate of root regeneration and their number per explant proved to be variable depending on particular medium treatment (Fig. 2). Although the first adventitious roots were noticed after 12 days of cultivation on *MSR* medium, during the first 4 weeks, the formation of new roots on applied medium was slow (average increment about 3). Moreover, in the 7th week, microplantlets in this treatment stopped producing new roots which reached the highest value equal to 28. Therefore, the microplants obtained on *MSR* medium were excluded from the scope of presently reported experiments. Instead, *1/2 MSR* medium stimulated rhizogenesis what was manifested by the constant increase of adventitious root number, especially between 32 and 36 day of observation (increment by 12). Due to this process at the end of those experimental stage, the largest number of regenerated roots, which exceeded 40 per explant, was observed on *1/2 MSR* medium. This value was 1.5 times higher than the value obtained in culture from *MSR* medium estimated in the same time. Thus, the convenient medium for permanent increase of root regeneration in *G. fastigiata* proved to be *1/2MSR.*

### Acclimatization of Microcuttings

Despite the protection with transparent containers, the strong turgor loss of shoots and their dying were observed just after 4 days of ex vitro cultivation. Finally, 46% of the regenerated plants survived during the step of greenhouse cultivation, and after 8 weeks, they were transplanted to a bigger pots filled with calamine substratum (Fig. 1d). In this experimental step, survival rate reached 100% and no negative influence of calamine substratum on *G. fastigiata* growth and development was noticed (Fig. 1e).

### In Vitro Selection and Evaluation of Plant Growth Parameters

**Fig. 3:**
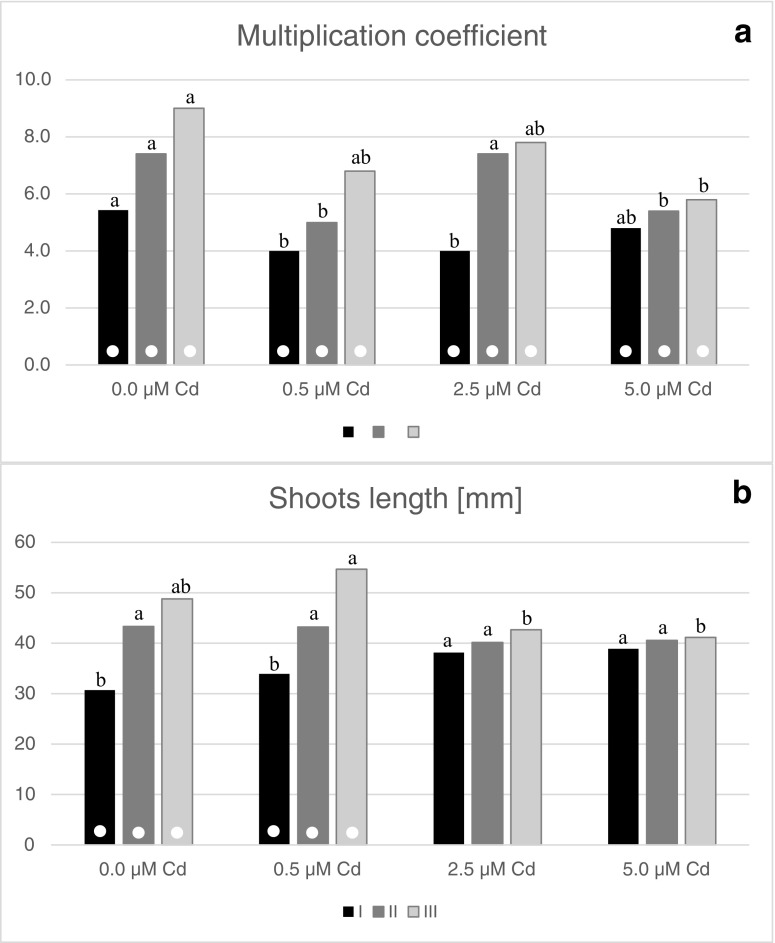
Changes in micropropagation efficiency of *G. fastigiata* on media supplemented with cadmium ions, evaluated after 8, 16, and 24 weeks of cultivation (I, II, III, respectively). Different letters within the following passage indicate means that are significantly different at *P* < 0.05. Dots indicate statistically significant correlation between the subsequent passage and particular growth parameter

During micropropagation step in the presence of heavy metals, the influence of lead nitrate on *G. fastigiata* growth was observed just after 3 weeks of 0.5 and 1.0 mM Pb^2+^ exposure. For these cultures, leaf necrosis and intensive anthocyanin discoloration were noticed (Fig. 1f–h). Similar symptoms of phytotoxic effects were found in cultures treated with 0.1 mM Pb(NO_3_)_2_; however, their reaction was delayed and noticeable after 8 weeks of cultivation. Finally, irrespective of the applied lead concentrations, the whole culture died. Thus, any biometric and biochemical measurements could not be performed. In the next experimental step, the reaction of shoots on the increasing concentration of cadmium chloride was investigated (Fig. 1i). After 8 weeks of cultivation, the micropropagation coefficient on medium supplemented with 0.5 and 2.5 μM CdCl_2_ amounted to 4 and was about 26% lower than in control culture. Moreover, the mean shoot length increased gradually with the increasing concentrations of cadmium ions from about 31 mm in the control treatment, through 34 mm in case of the lowest dose of Cd^2+^ to 39 mm in 2.5 and 5.0 μM Cd treatment (Fig. 3a). In turn, after 16 weeks of culture, the lowest values of micropropagation coefficient were noticed on media enriched with 0.5 and 5.0 μM Cd^2+^ (average MC = 5.2), while the highest number of regenerated shoots was obtained on medium without the addition of cadmium ions as well as on medium containing 2.5 μM Cd^2+^ (MC = 7.4) (Fig. 3b). After 16 weeks of cultivation, the length of propagated shoots varied from 40 to 43 mm. Nevertheless, these values were statistically insignificant (Fig. 3b). During the III passage, the micropropagation efficiency on Cd-supplemented media ranged from 5.8 to 7.8 (Fig. 3b), and the highest shoots were regenerated under the influence of 0.5 μM CdCl_2_, while the lowest one was in 5.0 μM Cd-treated culture (Fig. 3b). Independently on the treatment, the multiplication rate increased with cultivation time, and the largest differences in the average number of shoots regenerated from one explant between the first and the last passage were found for control and 2.5 μM Cd-treated culture (3.6 and 3.8, respectively) (Fig. 3a). Similarly, the shoot length changed with the lapse of Cd exposure, and the largest variation of this parameter was noticed in cultures growing on medium supplemented with 0.5 μM CdCl_2_. The obtained results indicated a significant positive correlation between the subsequent passages and micropropagation coefficient, which for shoots cultivated in the presence of 0.5 and 2.5 μM CdCl_2_ was at the moderate level of *r* = 0.58, while for both untreated shoots and shoots treated with 5.0 CdCl_2_ was about 0.35 (Fig. 3a). The weak uphill relationship of 0.37–0.41 between the time culture and shoot length was also shown for control and 0.5 μM treated cultures (suppl. data).

The highest concentration of Cd^2+^ significantly affected shoots fresh weight which was about 15% lower than in other treatments, and reached approximately 1.1 g (Table 2). On the other hand, the content of dry matter in shoots differed between treatments, and the highest amount was noted in shoots from medium with 2.5 CdCl_2_, while the lowest one in control culture. The applied concentrations of cadmium chloride significantly influenced on rhizogenesis (Table 2). In Cd-treated cultures, the percentage of spontaneously rooted explants varied from 33 to 42% and was about two times lower than in untreated one. Additionally, the adventitious root number regenerated per one explant and root length were strongly inhibited on media containing Cd ions. The decrease in examined characteristics resulted in the reduction of root fresh and dry matter in comparison with their contents obtained on non-supplemented medium.

**Table 2:** The fresh and dry matter content of *G. fastigiata* shoots as well as rhizogenesis after 24 weeks of cultivation in the presence of cadmium ions

Cadmium treatment	Shoot fresh weight (g)	Shoot dry weight (% f.w.)	Rooted shoots (%)	No. of roots/microplant	Root length (mm)	Root fresh weight (g)	Root dry weight (% f.w.)
0.0 μM CdCl_2_	1.34 a^a^	9.38 b	85.71 a	12.40 a	25.92 a	0.414 a	14.42 a
0.5 μM CdCl_2_	1.33 a	9.95 b	42.85 b	6.80 b	19.20 b	0.018 b	7.38 c
2.5 μM CdCl_2_	1.32 a	11.15 a	33.50 c	6.00 b	17.64 b	0.031 b	9.62 b
5.0 μM CdCl_2_	1.16 b	10.26 ab	0.00	0.00	0.00	0.000	0.00

Means indicated by the same letter within the columns do not significantly differ at *P* < 0.05 according to Fisher’s test

^a^Values are means of three replicates

### Biochemical Analysis

**Fig. 4:**
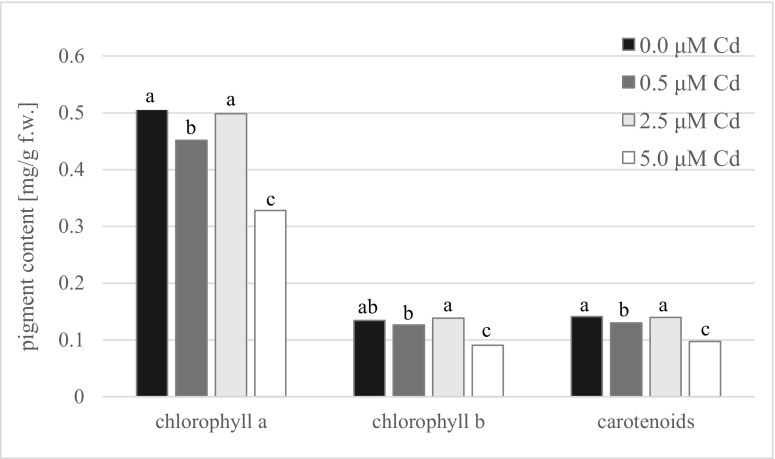
The content of photosynthetic pigments: chlorophyll *a*, chlorophyll *b*, and carotenoids in shoots of *G. fastigiata* cultured in the presence of various doses of cadmium chloride. Different letters indicate means that are significantly different at *P* < 0.05

**Fig. 5:**
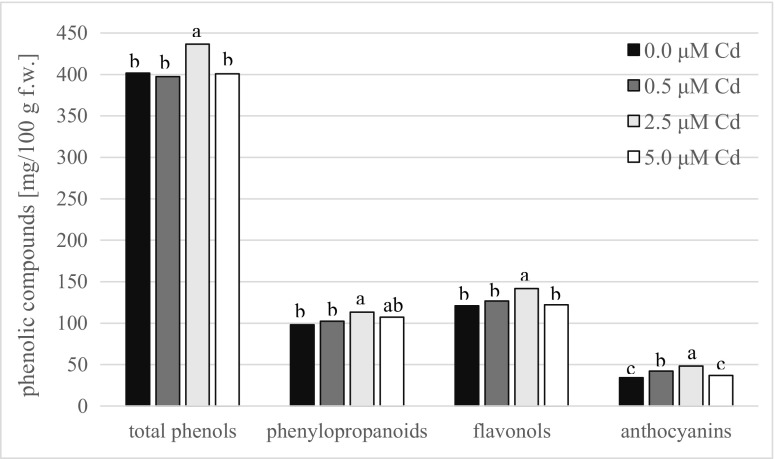
The concentration of phenolic compound in *G. fastigiata* shoots developed on media containing various doses of cadmium chloride. Different letters indicate means that are significantly different at *P* < 0.05

The concentration of photosynthetic pigments in shoots cultured in the presence of 2.5 μM CdCl_2_ was proved to be similar to that from medium without Cd ions and reached about 0.50 mg g^−1^ f.w. for chlorophyll *a*, 0.14 mg g^−1^ f.w. for chlorophyll *b*, and 0.13 mg g^−1^ f.w. for carotenoids (Fig. 4). The content of both chlorophyll types, as well as carotenoids, significantly decreased in shoots obtained on medium enriched with the highest dose of cadmium ions and reached the lowest values. In turn, 0.5 μM Cd-treated shoots contained intermediate pigment amount. The accumulation of all groups of phenolic compounds was significantly elevated in shoots of 2.5 μM Cd^2+^-treated line in comparison with other cultures (Fig. 5). Moreover, the differences in the level of phenols detected in control shoots as well as in shoots developed on media enriched with 0.5 and 5.0 μM CdCl_2_ were statistically insignificant and ranged from 397 to 401 mg for total phenols, from 98 to 107 mg for phenylopropanoids, and from 120 to 126 mg/100 g f.w. for flavonols, while in shoots developed on medium enriched with 2.5 μM CdCl_2_, their concentrations were significantly higher and got 436, 113, and 141 mg/100 g f.w., respectively. The most variable group of phenols was anthocyanins. Their accumulation in shoots cultivated in the presence of 2.5 μM CdCl_2_ was the highest (48 mg/100 g f.w.) and reached about 40% higher value than in control shoots and culture treated with 5.0 μM Cd^2+^.

## Discussion

In our previous studies, it was ascertained that among the species considered as a potential candidates for phytoremediation techniques, such calamine ecotype of *G. fastigiata* is an interesting object since it is a pioneer plant occurring spontaneously on Zn-Pb post-flotation waste tailings located in Olkusz Ore-bearing Region, and its importance for the reclamation of such deposits has been demonstrated in the field experiment (Muszyńska et al. 2015). In the present work, the tissue culture system competent to regenerate large amount of uniform plant material ready to be applied on heavy metal-polluted areas was elaborated. To our knowledge, it is the first report on in vitro propagation of *G. fastigiata* calamine ecotype and one of the few reports relating to the use of these techniques for multiplication of facultative metallophytes (Bidwell et al. 2001; Jack et al. 2005; Zheng et al. 2007; Muszyńska and Hanus-Fajerska 2017). In such experiments using of seeds collected from natural habitats to initiate in vitro culture is a common practice and was previously reported for others metallophytes like *Alyssum corsicum* (Babaoğlu Aydaş et al. 2013), *Plantago algarbiensis* and *P. almogravensis* (Gonçalves et al. 2009), or *Biscutella laevigata* (Hanus-Fajerska et al. 2012). Taking into account the culture condition optimized for economically important *Gypsophila* genus, various supplementation of MS medium were tested to elaborate the optimal medium composition for *G. fastigiata* calamine ecotype. In the study conducted by Zdraveva et al. (2015), it was found that the addition of IAA to growth medium was the most effective for secondary metabolite production of pharmaceutical importance in *G. trichotomas*, *G. altissima*, and *G. paniculata* shoot and callus cultures. In turn, according to the protocol proposed by Han et al. (1991), the use of MS medium containing BAP (2.0 mg L^−1^) and NAA (0.05 mg L^−1^) for establishment of shoot cultures of *G. paniculata* gave the best results. Likewise, Lee and Bae (1999) reported that the same cytokinin, but applied in lower concentrations (0.2 mg L^−1^ BAP), in the combination with NAA (0.1 or 0.2 mg L^−1^) were suitable for indirect regeneration of this species from apical buds. The positive effect of BAP (1.0 mg L^−1^) and NAA (0.2 mg L^−1^) on *G. paniculata* apical and axillary bud cultures was also shown in the study of Rashid et al. (2012). Although these growth regulators intensively stimulated the multiplication of *G. paniculata*, their application for culture of *G. fastigiata* calamine ecotype did not bring satisfactory results. In case of tested specimens, the best growth parameters were obtained on MS medium supplemented with 1.0 mg L^−1^ 2iP and 0.2 mg L^−1^ IAA. In order to initiate root regeneration, modified MS medium with reduced macro- and micronutrient by half and enriched with 1 mg L^−1^ IAA is proposed. The transfer of microplants to ex vitro condition proved to be an essential step that allowed to verify the usefulness of in vitro technique for effective propagation of tested genotype. Albeit the frequency of plants which stood the adaptation period was slightly lower than 50%, all of them survived on calamine substratum. It might suggest that the optimized micropropagation scheme provides the ex situ conservation options of this valuable plant species. Moreover, from 30 of aseptic *Gypsophila* seedlings, it was easily obtained about 200 of plantlets in 4 months. Therefore, the described protocol allows to regenerate a large number of plant material with intention to direct introduction on areas contaminated with heavy metals.

The optimization of reliable micropropagation protocol is a prerequisite step before conducting any in vitro selection experiments. The examined doses of lead nitrate applied to the medium for clonal propagation of *G. fastigiata* calamine ecotype induced growth disturbances and finally contributed to shoot culture death. Similarly, Wójcik and Tukiendorf (2014) have noted that lead nitrate even at the concentration of 30 μM negatively influenced on growth of another metal-tolerant species from Caryophyllaceae family, *Dianthus carthusianorum*, and its root development was totally inhibited in the presence of 850 μM Pb(NO_3_)_2_. The root system plays an important role in heavy metal detoxification. Many authors have reported that the endodermis with Casparian strips and pericycle cells have specific properties enabling them to block a symplastic transport of water and ions, and thus constitute a significant barrier to heavy metal translocation to aboveground parts of plant (Załęcka and Wierzbicka 2002; Baranowska-Morek and Wierzbicka 2004; Fernández et al. 2014; Wójcik and Tukiendorf 2014). In the present study, regardless of applied lead concentration, the regeneration of roots in *G. fastigiata* specimens was not observed. Probably, toxic ions could easily penetrate to the shoots without necessity to overcome root barrier what adversely affected plant metabolism and resulted in shoot culture dying. Thus, there is a need to verify if in the presence of fully expanded root system the negative effects of lead on shoot growth and development would be manifested. On the contrary, the proliferative shoot cultures of *G. fastigiata* were established on all media containing cadmium chloride and irrespective of Cd treatment obtained shoots were viable, with neither chlorotic, nor necrotic spots on leaves. What is more, the multiplication coefficients as well as shoot length increased on all tested media, suggesting that plants growth was not inhibited by cadmium. In order to better understand the mechanism of cadmium tolerance, several biochemical analyses were performed. The analysis of photosynthetic pigment content considered as an important indicator of heavy metals stress was significantly higher in shoots treated with 2.5 μM CdCl_2_ in comparison with other treatments and similar to control one. Other studies have also reported that chlorophyll level in leaves of tolerant plants does not change or ever increases in the presence of heavy metals (Burzyński and Buczek 1994; Dezhban et al. 2015). Taking into account fact that cadmium ions can negatively influence on plant organisms, the results of our study insinuate that selected line of *G. fastigiata* tolerant to Cd ions was obtained. Adaptation to Cd could be attributed to enhanced synthesis of phenolic compounds. These diverse secondary metabolites are involved in antioxidative defense systems that can protect cells from oxidative damage and scavenge harmful reactive oxygen species (ROS) commonly generated in the presence of elevated concentration of metallic elements (Lavid 2001a; Sharma et al. 2012; Wiszniewska et al. 2017). Moreover, phenolic compounds possess high ability to chelate metal ions and therefore participate in maintenance of homeostasis under heavy metal stress (Lavid et al. 2001b; Michalak 2006). In our study, the highest accumulation of all groups of phenols was observed in the best growing shoots cultivated on medium containing 2.5 μM CdCl_2_. Thus, one of the possible defense mechanism against cadmium stress in *G. fastigiata* calamine ecotype might be associated with enhanced accumulation of phenylopropanoinds. They are involved in lignin synthesis and its deposition within the cell walls what enable the formation of thicker and more effective mechanical barrier against the penetration of heavy metal ions inside the cell (Michalak 2006; Maestri et al. 2010). Such increase in lignin content correlated with the increase activity of phenylalanine ammonia-lyase activity (PAL, syn. EC); a key enzyme in phenylopranoid metabolism was observed in roots of *Glycine max* exposed to cadmium and lead (Pawlak-Sprada et al. 2011) or in *Matricaria chamomilla* under the influence of cadmium and zinc (Kováčik and Klejdus 2008). In turn, flavonols and anthocyanins, classified as flavonoids, are able to stabilize cell membranes by binding to their phospholipids and thus decreasing membrane fluidity (Michalak 2006; Karuppanapandian et al. 2011; Biesiada and Tomczak 2012). According to Verstraeten et al. (2003) and Arora et al. (2000), such membrane modification might hinder the diffusion of free radicals what reduce membrane damage resulted from lipid peroxidation. Thus, it is highly probable that the mechanism of heavy metal detoxification in selected Cd-tolerant line of *G. fastigiata* is based on the existence of extracellular barriers restricted ions penetration into the cytoplasm. In the course of present experiment, such strategy of metal detoxification could not prevent entering of Pb ions. The lead is usually excluded in roots, and obtained shoots deprived roots could not activate this defensive mechanism. As a result, they were directly exposed to highly elevated, phytotoxic levels of this element. Moreover, the applied doses of lead nitrate were very high and several times exceeded the concentration of soluble form of this element identified in the calamine substrate (Muszyńska et al. 2013, 2017). The above mentioned issues could have a significant impact on obtained results. Therefore, the restoration potential of *G. fastigiata* should be confirmed in the field experiment.

To conclude, in the present study, the efficient protocol of clonal propagation under in vitro conditions of valuable *G. fastigiata* population has been described for the first time*.* It is perennial herb with a deep root system which grows in the dry grassland deficient in nutrients. These features make *G. fastigiata* a potential candidate for application in rehabilitation schemes and also in management of not reclaimed wastes created as a result of Zn-Pb ore processing. Therefore, the experiments with the use of tissue culture techniques, which allow to obtain a great deal of regenerants with potential to stabilize toxic metallicolous wastes, are worth to be undertaken. Our study demonstrated that the in vitro derived plants are able to survive and develop on calamine substrate. Interestingly, the addition of lead nitrate to the propagation medium resulted in culture growth disturbances probably due to the lack of spontaneous rhizogenesis during presented experimental set. Thus, future research should focus on the role of root system in defense mechanism against Pb toxicity in tested species. On the contrary, it was found that cadmium supplementation might significantly increase multiplication efficiency and physiological condition of cultivated microplantlets. The strategy of *G. fastigiata* survival in the contaminated medium based on enhanced accumulation of phenolic compounds. The highest concentration of these secondary metabolites was observed in the best growing shoots cultivated on medium containing 2.5 μM CdCl_2_. It may implicate that we have obtained line tolerant to cadmium. However, future investigation into its exploitation in the revitalization of urban areas would be valuable.

## Electronic supplementary material

Esm 1 (DOCX 18 kb)
