# Supplementary material for: Differential Tolerance to Lead and Cadmium of Micropropagated Gypsophila fastigiata Ecotype
Source: Water Air Soil Pollut. 2018 Jan 25;229(2):42. doi: 10.1007/s11270-018-3702-8 (PMC5785615; doi:10.1007/s11270-018-3702-8)
Supplement: Supplementary file 1 — (DOCX 18 kb) [file 11270_2018_3702_MOESM1_ESM.docx]

Supplementary data:

The correlation coefficients (r) between the studied parameters of *G. fastigiata* cultures treated with cadmium ions with the p value. Marked values are statistically significant
at p <0.05.

|  |  |  | **Passage** | **No. shoots/ explant** | **Shoots length** |
| --- | --- | --- | --- | --- | --- |
| **0.0 μM CdCl_2_** | **Passage** | r |  | 0.3550 | 0.3718 |
|  |  | p |  | 0.000 | 0.000 |
|  | **No. shoots/ explant** | r | 0.3550 |  | 0.1500 |
|  |  | p | 0.000 |  | 0.118 |
|  | **Shoots length** | r | 0.3718 | 0.1500 |  |
|  |  | p | 0.000 | 0.118 |  |
| **0.5 μM CdCl_2_** | **Passage** | r |  | 0.5728 | 0.4105 |
|  |  | p |  | 0.000 | 0.000 |
|  | **No. shoots/ explant** | r | 0.5728 |  | 0.2578 |
|  |  | p | 0.000 |  | 0.016 |
|  | **Shoots length** | r | 0.4105 | 0.2578 |  |
|  |  | p | 0.000 | 0.016 |  |
| **2.5 μM CdCl_2_** | **Passage** | r |  | 0.5984 | 0.1355 |
|  |  | p |  | 0.000 | 0.143 |
|  | **No. shoots/ explant** | r | 0.5984 |  | 0.0373 |
|  |  | p | 0.000 |  | 0.689 |
|  | **Shoots length** | r | 0.1355 | 0.0373 |  |
|  |  | p | 0.143 | 0.689 |  |
| **5.0 μM CdCl_2_** | **Passage** | r |  | 0.3497 | 0.0940 |
|  |  | p |  | 0.001 | 0.404 |
|  | **No. shoots/ explant** | r | 0.3497 |  | 0.1860 |
|  |  | p | 0.001 |  | 0.096 |
|  | **Shoots length** | r | 0.0940 | 0.1860 |  |
|  |  | p | 0.404 | 0.096 |  |
